# The Transcriptome of Compatible and Incompatible Interactions of Potato (*Solanum tuberosum*) with *Phytophthora infestans* Revealed by DeepSAGE Analysis

**DOI:** 10.1371/journal.pone.0031526

**Published:** 2012-02-06

**Authors:** Gabor Gyetvai, Mads Sønderkær, Ulrike Göbel, Rico Basekow, Agim Ballvora, Maren Imhoff, Birgit Kersten, Kåre-Lehman Nielsen, Christiane Gebhardt

**Affiliations:** 1 Max-Planck Institute for Plant Breeding Research, Cologne, Germany; 2 Department of Biotechnology, Chemistry and Environmental Engineering, Aalborg University, Aalborg, Denmark; 3 Max-Planck Institute for Molecular Plant Physiology, Potsdam, Germany; University of Leeds, United Kingdom

## Abstract

Late blight, caused by the oomycete *Phytophthora infestans*, is the most important disease of potato (*Solanum tuberosum*). Understanding the molecular basis of resistance and susceptibility to late blight is therefore highly relevant for developing resistant cultivars, either by marker-assissted selection or by transgenic approaches. Specific *P. infestans* races having the *Avr1* effector gene trigger a hypersensitive resistance response in potato plants carrying the *R1* resistance gene (incompatible interaction) and cause disease in plants lacking *R1* (compatible interaction). The transcriptomes of the compatible and incompatible interaction were captured by DeepSAGE analysis of 44 biological samples comprising five genotypes, differing only by the presence or absence of the *R1* transgene, three infection time points and three biological replicates. 30.859 unique 21 base pair sequence tags were obtained, one third of which did not match any known potato transcript sequence. Two third of the tags were expressed at low frequency (<10 tag counts/million). 20.470 unitags matched to approximately twelve thousand potato transcribed genes. Tag frequencies were compared between compatible and incompatible interactions over the infection time course and between compatible and incompatible genotypes. Transcriptional changes were more numerous in compatible than in incompatible interactions. In contrast to incompatible interactions, transcriptional changes in the compatible interaction were observed predominantly for multigene families encoding defense response genes and genes functional in photosynthesis and CO_2_ fixation. Numerous transcriptional differences were also observed between near isogenic genotypes prior to infection with *P. infestans*. Our DeepSAGE transcriptome analysis uncovered novel candidate genes for plant host pathogen interactions, examples of which are discussed with respect to possible function.

## Introduction

The oomycete *Phytophthora infestans* causes late blight disease in Solanaceous plants, particularly in potato (*Solanum tuberosum*), tomato (*Solanum lycopersicum*) and their wild relatives. In potato plants, *P. infestans* attacks foliage, stems and tubers. When not controlled, late blight epidemics can completely destroy crop yield [1]. Late blight is therefore world wide the most important disease in potato cultivation. The control by pesticides increases considerably the production costs and can lead to the evolution of resistant *P. infestans* strains. Improving the genetic resistance of potato to late blight is therefore a long standing breeding goal, which is paralleled by numerous research efforts to describe, understand and manipulate the genetic basis of resistance. Genetic resistance to *P. infestans* has been discovered in many wild potato species and was introgressed during the last century into the cultivated potato primarily from the Mexican wild species *S. demissum* [2]. Host resistance to specific races of *P. infestans* is conferred by single, dominant *R* genes, which recognize the corresponding avirulence (*Avr*) gene of *P. infestans* and trigger a defense response manifesting itself in local cell death (hypersensitive resistance, HR), thereby arresting pathogen growth. In this case the host plant and the pathogen are incompatible (incompatible interaction). In recent years, several *R* and *Avr* genes have been cloned from potato and *P. infestans*, respectively, and functionally characterized [3], [4], [5], [6], [7], [8], [9], [10], [11], [12]. All potato *R* genes for resistance to *P. infestans* characterized so far are members of the CC-NBS-LRR type gene family [13], typically having a coiled coil (CC) domain, a nucleotide binding site (NBS) and a leucine rich repeat (LRR) domain. Pathogen recognition by an *R* gene is quite easily circumvented by mutations in the corresponding *P. infestans* avirulence gene, which enables the pathogen to successfully invade and colonize the host plant in a compatible interaction. The compatible interaction is not uniform. Depending on the genotype of the host plant, invasion, growth and sporulation of *P. infestans* progresses with variable efficiency and speed. This natural variation of the compatible interaction constitutes another type of resistance, which is controlled by multiple genetic and environmental factors and is called quantitative resistance [14]. The identification of the genes that underlay quantitative resistance is in its infancy. Genetic analyses suggest that ‘defeated’ *R* genes (*R* genes overcome by new races of *P. infestans*) are one component of quantitative resistance to late blight [14], [15], [16], [17]. Further candidates for quantitative resistance are genes functional in defense signaling [18], [19] and defense response genes [20].

The compatible and incompatible interaction of potato with *P. infestans* involves transcriptional activation or repression of a large number of genes. A fraction of these pathogenesis related genes has been characterized at the molecular level, including expression changes upon infection with *P. infestans*, initially on a gene by gene basis, for example [21], [22], [23], [24]. Then, PCR (polymerase chain reaction) based subtractive hybridization [25] made possible the parallel isolation of dozens known as well as novel cDNAs that were differentially expressed upon infection with *P. infestans*. Large DNA sequence databases facilitated the assignment of putative functions to differentially expressed cDNAs by sequence similarity [26], [27], [28], [29]. A further step towards monitoring the full potato transcriptome in response to *P. infestans* infection were hybridizations of microarrays [30] with cDNA probes from infected and non infected leaves, which yielded hundreds of known and new differentially expressed genes [31], [32], [33]. With the exception of the early papers analysing single gene expression in the same genotype after infection with virulent or avirulent strains of *P. infestans* [22], [23], [24], the recent high troughput studies do not allow a direct comparison of the compatible and incompatible interaction in the same genetic background, because either an incompatible or a compatible interaction was analysed for expression changes over an infection time course, or incompatible and compatible interactions were compared between different potato genotypes or even species. Either infection of the same genotype carrying an *R* gene with a virulent or avirulent *P. infestans* race, or infection of ‘near isogenic’ genotypes transformed with an *R* gene and comparison with the susceptible wild type should facilitate the detection of transcripts that are specifically up- or down regulated in the compatible or incompatible interaction, independent from the genetic background. Transcripts that are up- or down regulated in the compatible interaction are candidates for being involved in quantitative resistance and may be further analyzed, for example by association genetics [18] or gene silencing approaches [19].

Next generation sequencing technology [34] has opened new possibilities for de novo, comprehensive analysis of the transcriptome by serial analysis of gene expression (SAGE) [35]. DeepSAGE [36] is a further development of SAGE. Libraries of 21 base pairs DNA sequence tags are generated from poly-adenylated RNA and subjected to massive parallel sequencing. Ideally, all transcripts present in a biological sample are detected by specific sequence tags, the frequency of which is linearly related with the frequency of the transcript in the sample. Differentially expressed transcripts are identified by comparing the tag frequencies in libraries generated from two or more biological samples.

We used DeepSAGE technology to comprehensively capture and compare the transcriptomes of the compatible and the incompatible interaction between *S. tuberosum* and *P. infestans*. The transcriptome of the incompatible interaction was obtained from two independent transgenic lines carrying the *R1* gene for resistance to late blight in the same genetic background [6], whereas the transcriptome of the compatible interaction was obtained from the same untransformed genotype and further transgenic lines transformed with the empty vector or a paralogous member of the *R1* gene family of unknown function. Our analysis identified novel differential transcripts that might have a functional role during compatible or incompatible host-pathogen interactions.

## Results

### DeepSAGE libraries

We generated fourty four 3′ sequence tag libraries from five genotypes, three time points and three biological replicates (one tag library failed (ORF45_3dpi_rep3)) from total RNA extracted from leaf tissue of single plants. The tissue samples were collected 0, 1 and 3 days after infection of five nearly isogenic potato genotypes with a *P. infestans* race carrying the avirulence gene *Avr1*. Two independent transgenic Désirée lines (10-2/4 and 10-23/2) carrying a single insertion of the *R1* resistance gene represented the incompatible interaction, whereas untransformed Désirée (WT), a transgenic Désirée line carrying the empty vector (LV41) and a Désirée line transformed with a 10 kbp potato genomic fragment containing the *r1.1* gene (ORF45), represented the compatible interaction. The function of the *r1.1* gene is not known. It shares high sequence homology with *R1* but does not confer resistance to *P. infestans* carrying *Avr1* [6]. The ORF45 transgenic line contains one or more fragments of unknown size of the 10 kbp construct (see Materials and Methods). Deep sequencing of the 44 tag libraries resulted in 47.189809 million high quality reads. Removal of singleton sequences left 37.115611 million reads, corresponding to 30 859 unique 21 base pair sequence tags (unitags) (Table S1), which were further analysed.

**Figure 1:**
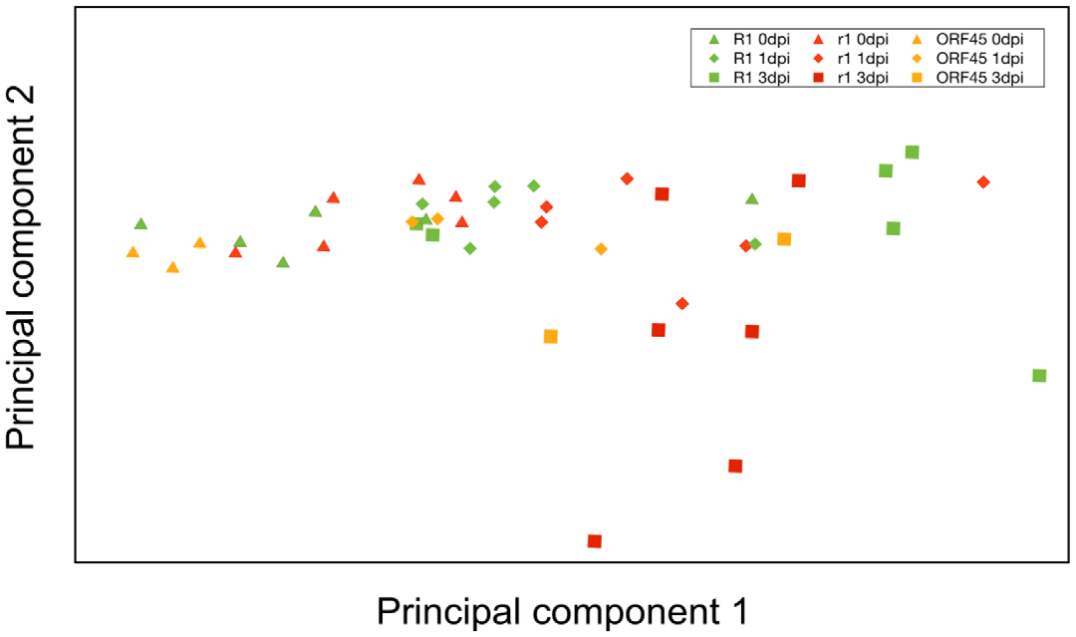
Biplot of the principal components one and two after principal component analysis based on the transcriptome data of 44 samples.

Principal component analysis (PCA) of the 44 samples (Figure 1) showed clustering according to the infection time course along the axis of the first principal component. At 0 dpi (days post infection) most samples clustered separately from the samples at 1 dpi and 3 dpi, irrespective of genotype. Nevertheless, even at 0 dpi dispersion occurred along the axis of the first principal component, indicating considerable transcriptome variation among near isogenic, uninfected plants. The largest differentiation between the samples occurred at 3 dpi. Samples having *R1* (incompatible interaction) were separated from the *r1/ORF45* samples (compatible interaction) at 3 dpi. However differences within the *R1* and *r1/ORF45* samples were as large as the differences between the two groups. A clear grouping of samples according to genotype and time points after infection was therefore not observed.

To facilitate data analysis and to improve the reliability of differential tag identification, we combined the data from the two incompatible (*R1*) lines 10-2/4 and 10-23/2 and those from the compatible (*r1*) lines WT and LV41 (3 biological replicates each) in two genotypic groups named R1 and r1, respectively. Pair wise comparisons between time points and between incompatible and compatible genotypes *R1* and *r1* were therefore based on tag libraries of six independent biological samples (two genotypes, three biological replicates) per time point and group (Table 1). The transcriptome data of line ORF45 were analysed separately from the R1 and r1 groups. The statistics of line ORF45 was based on two to three biological replicates of a single transgenic line (Table 1). The average library size per grouped samples ranged from 764873 to 1 406649 sequence tags and the number of unitags per library from13288 to 18919 (Table 1).

**Table 1:** Summary of the 3′sequence tag libraries used for pair wise comparisons.

Genotypic group	Time point^4^	No. of samples	Average library size	SD library size	Average number of unitags	SD unitag number	Type of interaction^5^
R1^1^	0 dpi	6	982 739	351 930	16 093	4 530	incompatible
	1 dpi	6	1 249 088	298 093	18 919	3 926	incompatible
	3 dpi	6	994 582	305 624	16 172	5 150	incompatible
r1^2^	0 dpi	6	884 486	252 353	13 288	4 265	compatible
	1 dpi	6	1 034 652	433 311	15 003	3 063	compatible
	3 dpi	6	1 115 435	142 596	15 937	3 978	compatible
ORF45^3^	0 dpi	3	1 291 402	527 049	16 731	6 238	compatible
	1 dpi	3	1 406 649	598 539	17 426	5 913	compatible
	3 dpi	2	764 873	537 101	14 994	10 862	compatible

1 Combination of the Désirée transgenic lines 10-2/4 and 10-23/2 containing the *R1* gene.

2 Combination of untransformed Désirée (WT) and Désirée transgenic line LV41.

3 Désirée transgenic line, transformed with a 10 kbp genomic fragment containing the *r1.1* gene (ORF45).

4 Time after inoculation in dpi (days post inoculation) when leaf tissue was sampled.

5 Type of interaction between the host and the pathogen. ‘Incompatible’ indicates a hypersensitive resistance response whereas ‘compatible’ indicates a successful colonization of the host tissue.

**Figure 2:**
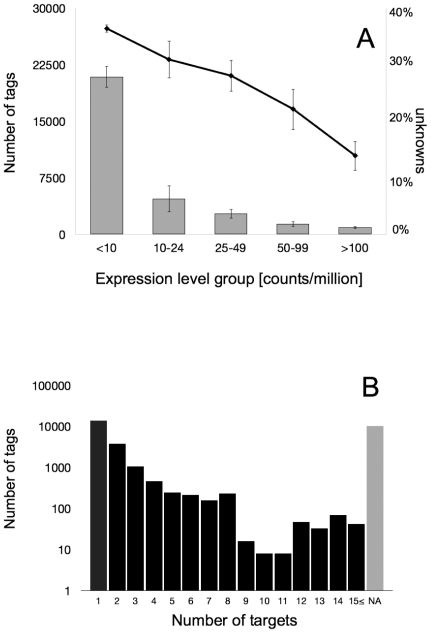
Categories of tag frequencies, fraction of unknown tags per category and number of tags matching one or more target sequences. (**A**) Histograms of the average tag frequencies. The curve above the histogram in **A** shows the average proportion (y-axis on the right) of unknown tags in the five frequency classes. (**B**) The number of tags matching from 1 to more than 15 different target sequences. NA: Number of tags with no match.

The majority of the 30859 unitags showed low expression levels. 20929 unitags had an average expression level of less than 10 counts/million and only 867 unitags were detected with more than 100 counts/million (Fig. 2A). 20470 unitags (66.33%) matched to expressed potato genes (sequence targets) available in public databases. The remaining 10389 unitags were not represented in the public potato unigene collections. The percentage of unknown unitags decreased with increasing expression level (Fig. 2A). In the lowest expression class (<10 counts/million), 36% of the unitags were unknown compared to 14% in the highest expression class (>100 counts/million). The specificity of the tag annotation was assessed by counting the number of unitags that matched to one or more target sequences. 14065 (45.58%) and 3789 (12.28%) unitags matched to one or two target sequences, respectively. The remaining 2616 annotated unitags (8.48%) matched to three or more target sequences (Fig. 2B). In many cases, multiple unitags of highly variable abundance matched to the same target sequence, indicating expression of multiple gene copies and/or allelic variants of the target sequence in the sample. An unknown portion of these tags, particularly low abundant ones, are also likely artefacts resulting from incomplete restriction digestion during library construction or sequencing errors. Discounting redundant tags, the matching unitags corresponded to 12312 target sequences or expressed *Solanum tuberosum* genes. One hundred and thirty nine unitags matched the genomic sequence of *Phytophthora infestans* strain T30-4 [37]. As some of these tags were also present in the 0 dpi samples, they likely correspond to genes conserved between *P. infestans* and other microorganisms, which colonized the sampled leaves.

### Standard defense responses

**Figure 3:**
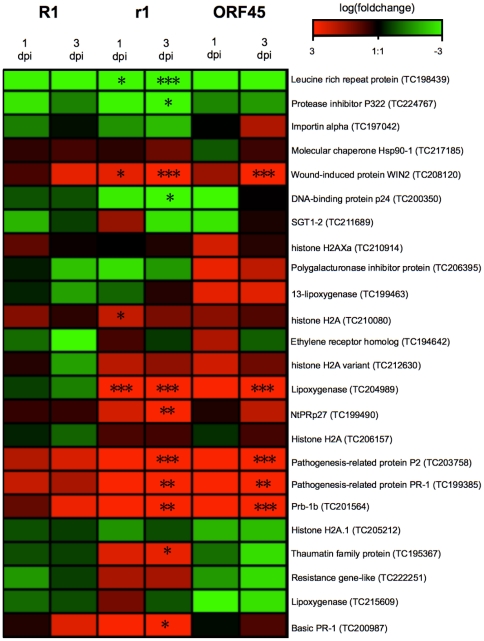
Differential expression of known defense response genes in compatible and incompatible interactions analysed by hierarchical cluster analysis. The heat map of the log-fold change values of sample groups R1, r1 and ORF45 at 1 dpi and 3 dpi versus 0 dpi is shown. Unitags matching to twenty four transcripts were selected based on allocation to the gene ontology term “defense response” (GO:0006952). The annotation and the corresponding unigene number are shown on the right. Significance of the pair wise comparisons between R1, r1 and ORF45 sample groups is indicated by * (FDR≤0.05), ** (FDR≤0.01) and *** (FDR≤0.001).

A number of genes are known to be characteristically up or down regulated upon pathogen infection [38]. In order to verify infection of the sampled leaf tissues, the data were searched for known expression signatures for successful infection by the pathogen. Twenty four unitags grouped under the gene ontology (GO)-term “defense response” (GO:0006952) were extracted from the data set, which matched most reliably defense response genes according to the annotation of the Potato Gene Index by the Dana Faber Cancer Institute. The expression levels of these genes at 1 dpi and 3 dpi as compared to 0 dpi were analysed by hierarchical clustering of the corresponding tags (Fig. 3, Table S2). Twenty two unitags showed a log-fold expression change of at least 2 at 1 dpi and/or 2 dpi compared to 0 dpi and twelve of those tags showed significant (FDR≤0.05) changes, primarily during the compatible interaction at 3 dpi (r1 and ORF45). This set of tags included well known pathogenesis related (PR) genes, the expression of which is up regulated upon pathogen attack. Certain ‘defense response’ tags appeared to be up regulated in the compatible but not in the incompatible interaction (histone 2A variant (TC218608), lipoxygenase (TC204989), NtPRp27 (TC199490)). Variation between the expression patterns of the r1 genotypes and ORF45 was also observed.

### Differential expression in compatible and incompatible interactions

**Figure 4:**
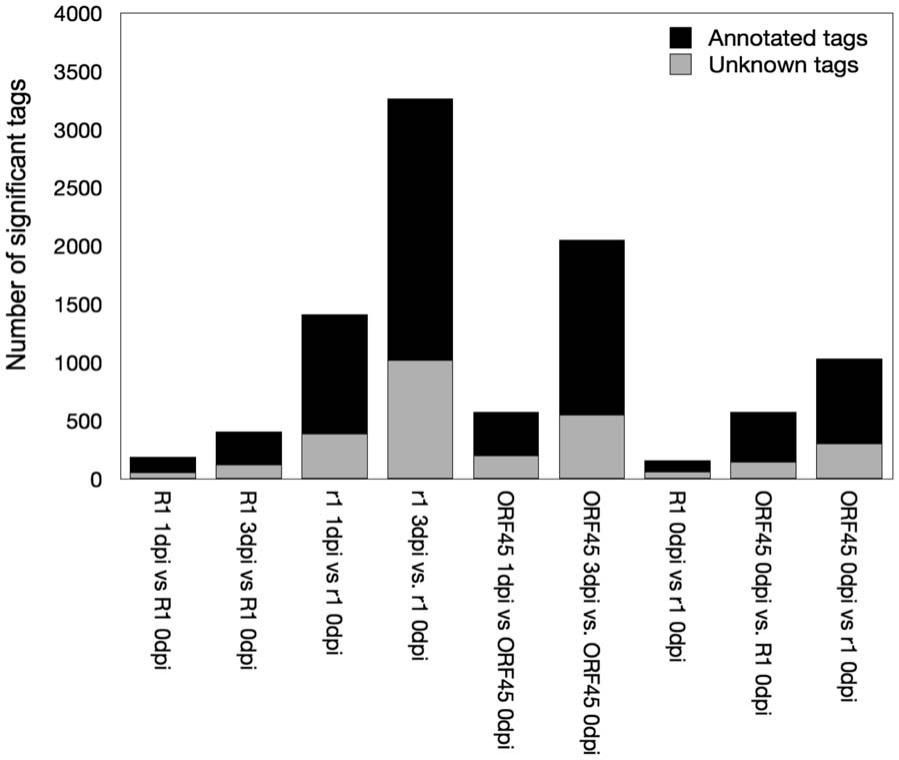
Number of tags showing significant expression changes (FDR≤0.05) in nine pair wise comparisons. The grey bar indicates the number of unknown tags. Incompatible (R1) and compatible (r1, ORF45) interactions at 1 dpi and 3 dpi were compared with 0 dpi. At 0 dpi, genotypic groups were compared: R1 with r1 and ORF45, and ORF45 with r1.

We performed nine pair wise comparisons between expression tag libraries, two comparisons each for the genotypic groups R1, r1 and ORF45 over the infection time course and three comparisons among the genotypic groups at 0 dpi (Fig. 4). Transcriptional changes upon infection were more numerous in the compatible as compared to the incompatible interaction. Three days after infection with *P. infestans*, a five to eight-fold higher number of differentially expressed unitags (false discovery rate FDR<0.05) was observed for the compatible interactions (r1 and ORF45) as compared to the incompatible interactions (R1) (Fig. 4). The highest number of 3265 differential unitags was found in the r1 samples at 3 dpi versus 0 dpi.

**Figure 5:**
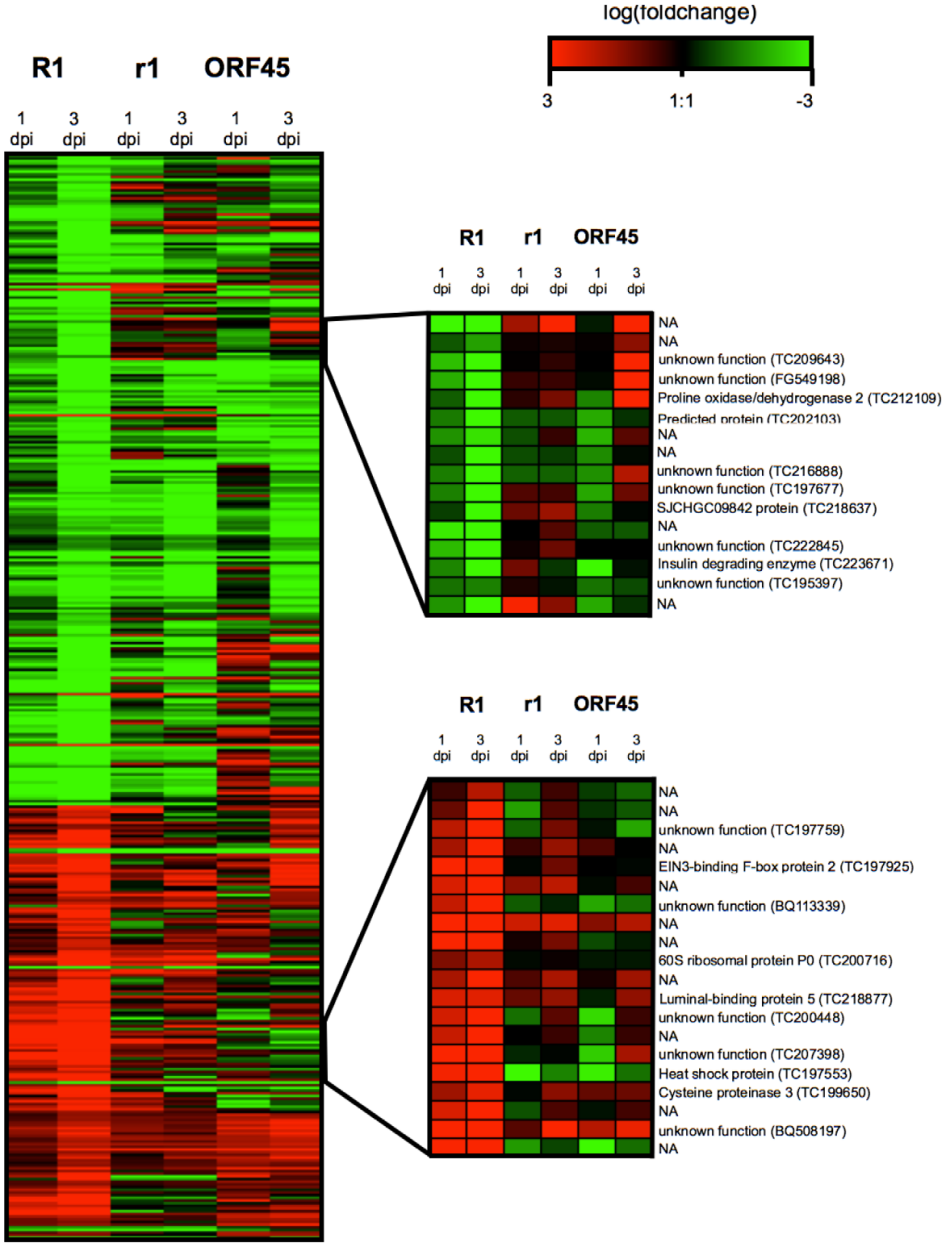
Heat map of the log-fold change values of sample groups R1, r1 and ORF45 at 1 dpi and 3 dpi compared to 0 dpi. Shown is the result of a hierarchical cluster analysis using 406 unitags with significant expression changes in the incompatible interactions (R1) at 3 dpi compared to 0 dpi. Two regions with preferential up or down regulation in the incompatible interaction are shown enlarged with the tag annotation given on the right side. NA: Tag with no matching target sequence.

Hierarchical cluster analysis of 406 unitags with significant expression changes in the incompatible interactions (R1) at 3 dpi compared to 0 dpi showed similarities and dissimilarities with compatible interactions (r1 and ORF45) (Fig. 5). Some clusters showed specificity for incompatible interactions. Two examples are enlarged in Figure 5. The first cluster contained 16 unitags, mostly with unknown function, which were all clearly down regulated in the *R1* transgenic plants but less so in the *r1* plants. The ORF45 transgenic line did not show a consistent expression pattern in this group of tags. One of the most interesting members in this group is a gene encoding a putative SJCHGC09842 protein. A member of this class of proteins was originally described in *Schistosoma japonicum* and shows structural similarities to the Cathepsin L class [39]. This gene was down regulated in R1 and up regulated in r1 plants. Transcript changes of this gene in the ORF45 plants were similar as in R1 plants at 1 dpi but at 3 dpi the expression increased to the initial level (Fig. 5). The second cluster contains 20 tags, also mostly unknown, which show primarily up regulation in the *R1* transgenic lines. Three tags in this group are remarkable, because they responded in the compatible interaction in the opposite direction. The first matched to a heat shock protein (TC197553), the second to a gene with unknown function (BQ113339) and the third lacked any annotation.

**Figure 6:**
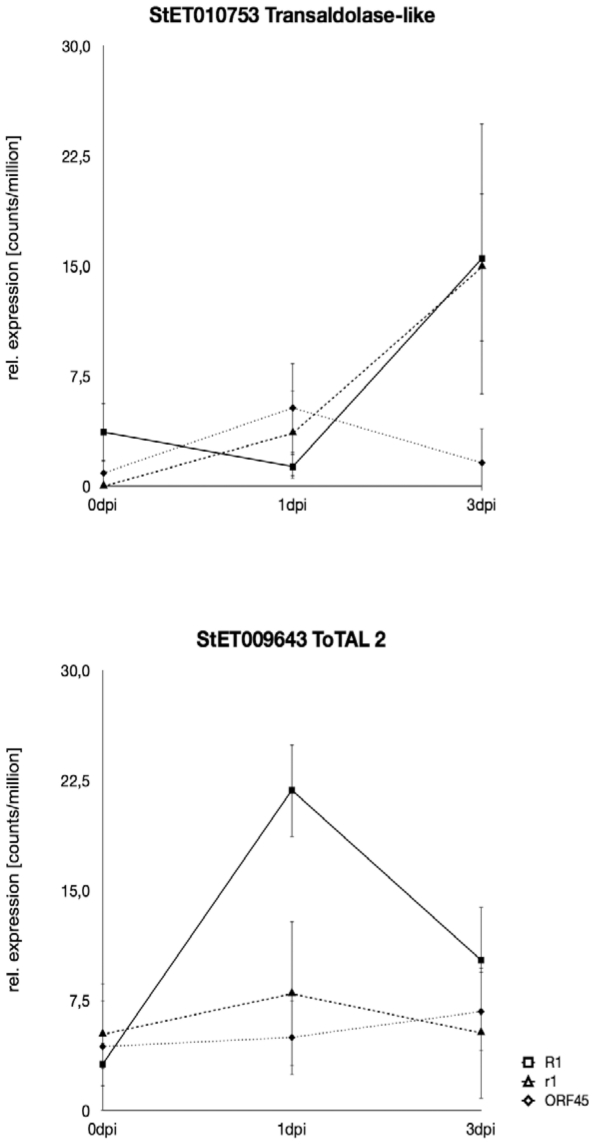
Expression patterns of tags StET010753 and StET009643 matching the tomato transaldolase isoforms ToTAL1 (PotTAL1) and ToTAL2, respectively. Tag StET10753 is weakly up regulated in the R1 and r1 plants but not in the ORF45 plants at three days post inoculation. Tag StET009643 shows clear up regulation only in the R1 plants one day after inoculation. Transcript levels are shown as mean tag counts/million of six (R1 and r1 plants), three (ORF45 plants at 0 dpi and 2 dpi) and two (ORF45 plants at 3 dpi) independent samples. Bars indicate the standard deviation according to the Poisson distribution.

To address the question, how many and which unitags were preferentially up or down regulated in either compatible or incompatible interactions, we filtered the data on the one hand for potato tags (excluding tags derived from *P. infestans*) that showed significant expression differences only in the *R1* transgenic lines at 1 dpi and/or 3 dpi when compared to 0 dpi (incompatible interactions). Three hundred ninety unitags fulfilled these criteria (Table 2, Table S3). Twenty eight tags were differentially expressed at both 1 dpi and 3 dpi. The 267 known tags matched to 240 different target sequences. Ten target sequences matched to multiple tags. Around one quarter of the tags had annotations that suggest a putative function. Among those were the tags with the lowest FDR values, which matched to the accessions TC207935 (tag StET010841 down regulated at 3 dpi, FDR = 5,85×10^−16^) annotated as fibrillin 8, CV474958 (tag StET002320 up regulated at 3 dpi, FDR = 1.86×10^−13^) annotated as a putative dihydroorotate oxidoreductase, and TC196885 (tag StET009643 up regulated at 1 dpi, FDR = 2.62×10^−6^, Fig. 6), encoding a transaldolase (EC2.2.1.2).

**Table 2:** Rational and summary of the comparisons between compatible and incompatible interactions over the infection time course, and between genotype groups R1, r1 and ORF45 at 0 dpi.

Tag data filtering conditions	No. of differential tags	No. of unknown tags	No. of up regulated tags	No. of down regulated tags	No. of target sequences
FDR<0.05 in R1, FDR>0.05 in r1 and ORF45, comparing 1 dpi and 3 dpi with 0 dpi	390	123	204	186	240
FDR<0.05 in r1 and ORF45, FDR>0.05 in R1, comparing 1 dpi and 3 dpi with 0 dpi	796	178	432	364	220
FDR<0.05 in R1, r1 and ORF45, comparing 1 dpi and 3 dpi with 0 dpi	28	7	3	25	13
FDR<0.05, comparing R1 with r1 and ORF45 at 0 dpi	7	2	2	5	5
FDR<0.05 in ORF45, FDR>0.05 in r1 and R1, comparing 1 dpi and 3 dpi with 0 dpi	1471	463	793	678	744
FDR<0.05, comparing ORF45 with r1 and R1 at 0 dpi	199	46	194	5	65

Filtering the data on the other hand for unitags that were differentially expressed only in the r1 and ORF45 plants at 1 dpi and/or 3 dpi when compared to 0 dpi (compatible interactions), resulted in 796 potato tags, of which 178 were unknown (Table 2, Table S4). The 618 known tags matched to 220 different target sequences. This was due to the fact that 50 (23%) target sequences were matched by multiple tags, indicating that some of these tags correspond to multigene families and/or multiple alleles. Multiple alleles are expected to occur in the tetraploid potato which is highly heterozygous. Among others, two functional categories were prominent among the sequences targeted by multiple unitags: defense response genes such as PR1 (TC199385), PR2 (TC203758), PR10 (TC198314) and chitinase (TC224221), all up regulated upon infection, and genes functional in photosynthesis and CO_2_ fixation such as components of photosystem I and II (e. g. TC197880, TC198430, TC199524, TC219799, TC213698), ferredoxin 1 precursor (TC212710), chlorophyll a–b binding protein (TC195879) and ribulose bisphosphate carboxylase small subunit (TC223622, TC201192), which were all down regulated (Table S4).

Only 28 tags were consistently up or down regulated in both compatible and incompatible interactions (Table 2, Table S5). The most conspicuous among those with meaningful annotation were protease inhibitors encoded by multigene families [40]. Two hundred and one tags were extracted from the whole data set that matched to 48 target sequences annotated as various protease inhibitors. After confirmation of the annotation by blasting the target sequences against the nucleotide sequence collection at NCBI (http://blast.ncbi.nlm.nih.gov/Blast), 150 tags matching 47 target sequences remained, which corresponded to at least 13 different types of protease inhibitors (Table S6). With the exception of serine protease inhibitors (TC206576, TC203946), members of all inhibitor types showed differential expression upon infection with *P. infestans*. Members of the following inhibitor families were down regulated in compatible and incompatible interactions, although less so in incompatible interactions: ‘Protease inhibitor 1’ (Pin1, e.g. TC221091, TC216360, CV503740, TC214045), ‘aspartic protease inhibitors 1 and 5’ (CV475336, TC197847), ‘cysteine protease inhibitor’ (e.g. TC222339, TC199588), ‘trypsin inhibitor’ (TC199575), ‘protease inhibitor 2 TR8 precursor’ (Pin2, TC205638) and ‘protease inhibitor isoform’ (TC223032). Interestingly, some inhibitors were predominantly up or down regulated in compatible interactions. Down regulated was ‘Probable protease inhibitor P322’ (e. g. TC224767, CV475377). Up regulated were ‘Metallocarboxypeptidase inhibitor’ (TC225685, TC222028, TC195941) and ‘ethylene-responsive proteinase inhibitor 1’ (TC219689) (Table S6).

### GO-terms over represented in compatible and incompatible interactions

**Figure 7:**
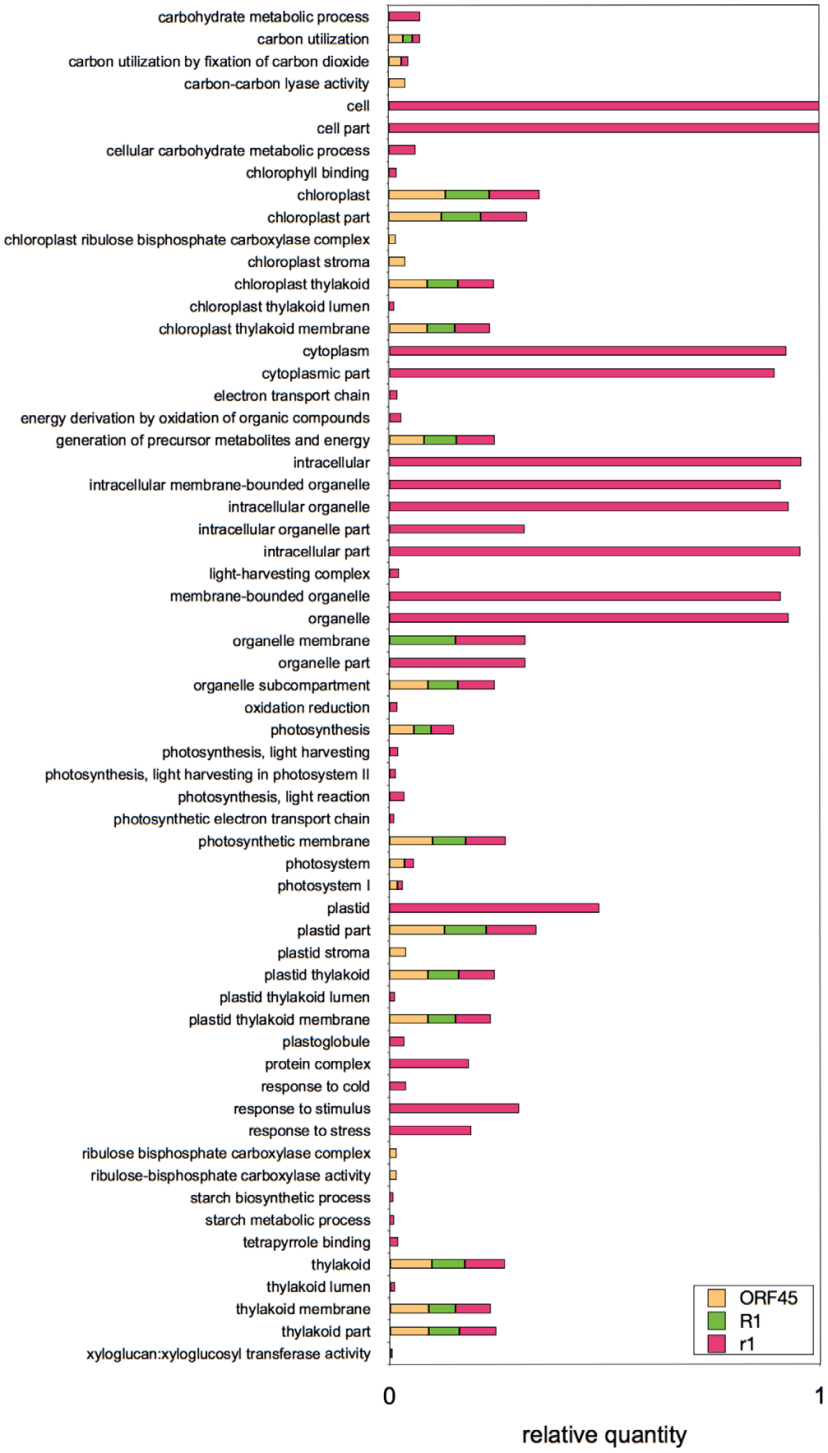
Histogram of significantly (FDR≤0.1) over represented gene ontologies (GO). The Tags differentially expressed three days after inoculation (3 dpi) in the R1, r1 and ORF45 sample groups were used for the analysis. The bars indicate relative tag quantity by determining the ratio between the representation of an ontology term and the total number of significant tags.

To obtain an overview on the processes which might be involved in the compatible and incompatible interactions, a GO (gene ontology)-term analysis was performed using those unitags, which were differentially expressed three days after inoculation (3 dpi) in the R1, r1 and ORF45 genotypic groups. Testing for over representation identified 61 significant GO-terms (Fig. 7). Fifteen GO-terms were over represented in all three genotypic groups R1, r1 and ORF45. They related to the photosynthetic apparatus, the utilization of carbon and the generation of precursor metabolites and energy. Thirty three GO-terms were exclusively over represented in the r1 plants (compatible interactions), including the terms ‘response to stress’, ‘response to cold’, ‘response to stimulus’ and ‘carbohydrate metabolic process’. In the ORF45 transgenic plants five terms were specifically over represented which were mainly connected to carbon fixation represented by terms such as “ribulose bisphosphate carboxylase activity” or “carbon-carbon lyase activity”. No GO-term occurred specifically in the R1 plants (incompatible interactions).

### Differential expression between genotypes at 0 dpi

The comparison of the genotypic groups R1, r1 and ORF45 with each other at 0 dpi should reveal expression differences, that either exist in these plants independent of infection with *P. infestans*, or are extremely rapid induced upon infection (within four minutes after inoculation, see Materials and Methods for sample preparation). Comparing the expression profiles at 0 dpi between the groups R1 and r1 resulted in 159 different tags (FDR<0.05), between R1 and ORF45 in 576 and between r1 and ORF45 in 1026 different tags (Fig. 4). The overlap between the three comparisons was small. When filtering the data further for tags with significant differences in both comparisons R1 versus r1 and R1 versus ORF45, only 7 tags remained (Table 2, Table S7). Except tag StET005879 (TC207421), which was annotated as a GRAS9 transcription factor, none of these tags had a meaningful annotation. Two unknown tags showed in *R1* transgenic plants higher expression than in both r1 and ORF45 plants, whereas the other five tags showed the opposite pattern, low expression in *R1* transgenic plants and up regulation in r1 and ORF45 plants.

### Differential expression in ORF45

The transgenic line ORF45 contains one or more fragments of unknown size from a 10 kbp construct containing the potato *r1.1* gene. Unitags differentially expressed only in ORF45 plants when compared with R1 and r1 plants might be connected to function, position and copy number effects of the transgenic DNA insertions in cv Désirée background. We filtered the data set for tags up or down regulated preferentially in ORF45 plants at 1 dpi and/or 3 dpi versus 0 dpi. This turned up 1471 unitags, of which 149 were differentially expressed at both 1 dpi and 3 dpi (Table 2, Table S8). The 1008 known tags matched to 744 different target sequences. Finally, we selected tags with significantly different expression levels in ORF45 when compared to both R1 and r1 plants at 0 dpi. This comparison resulted in 199 tags (Table 2, Table S9). With 5 exceptions, these tags were expressed at higher level in ORF45 plants than in both R1 and r1 plants. Eighty nine of these tags showed no changes over the infection time course, among those candidates such as SKP1-like protein (TC201682), EF hand family protein (TC200512), Cullin 3-like protein (TC198425), nuclear receptor GRF (CK252537) and LeArcA1 protein (TC197505). Due to multiple tags targeting the same sequence, the 152 annotated tags matched to 63 target sequences, many of which are annotated as genes operating in photosynthesis and carbon fixation, similarly as observed for the compatible interactions over the infection time course. Many of these tags were also differentially expressed during infection.

## Discussion

In this study, we used next generation sequencing of 44 DeepSAGE libraries to detect and quantify the transcripts present in potato leaves before and after infection with *P. infestans*. To eliminate effects of the genetic background on the leaf transcriptome, we used for infection experiments and sampling five near isogenic genotypes derived from cv Désirée, which differed only by the presence or absence of the *R1* resistance gene. The comparison of the interaction of potato with *P. infestans* at three infection time points was based on six and nine independent biological samples for the incompatible and the compatible interaction, respectively. This redundancy provided very stringent experimental conditions for detecting differentially expressed transcripts. The statistical treatment of comparisons between SAGE data from multiple samples such as described here is in an exploratory phase. Several test statistics have been proposed to handle comparisons of SAGE data, for example the G-test [41], a variation of Fisher's exact test [42], baySeq [43], DEGseq [44], DESeq [45] and edgeR [46]. After exploring the DeepSAGE data with the G-test, Fisher's exact test, baySeq (unpublished results) and edgeR, we found that edgeR, for the time being, was the most appropriate method when performing multiple sample comparisons. However, we observed that the test statistic does influence the results obtained. As there is no consensus yet on the optimal test statistic for identifying ‘true’ differences between tag counts, particularly those with low frequency, our extensive data set represents an excellent basis for method comparison and improvement.

Principal component analysis (Fig. 1) based on the tag data of all 44 samples, each representing several leaflets of a single plant, showed large variation of individual defense responses. Most samples showed clustering at 0 dpi, although less than it was expected for genetically nearly identical plants prior to infection. Dispersal of the samples along both axes was observed at 1 dpi and more so at 3 dpi, indicating significant changes of the transcript profiles, but without consistent grouping according to time point or interaction type. Looking at actual tag counts, large variation was observed between individual plants within the same group at the same time point. This indicates that the timing of the defense response varied considerably between individual plants, some responding faster than others. Many physiological and environmental factors can influence the timing of individual defense responses. One might be the movement in time and space of defense signals from the site of the initial physical contact between *P. infestans* zoospores and host cells and the neighboring cells. To avoid contamination of the transcriptome data with tag sequences originating from *P. infestans*, we sampled the tissue for transcriptome analysis from inoculated leaflets but excluding the infection points where the initial haustoria are established (see Materials and Methods). Variation of defense signaling could therefore have contributed to the individual variation of transcript profiles.

During the first three days after infection with *P. infestans*, the leaves are almost symptomless. Visible HR or sporulating leasons develop after four to six days. Infection was therefore monitored by extracting from the SAGE data a set of unitags which corresponded to genes known to be differentially regulated during the defense response of *Solanum tuberosum*. Most of these defense response tags showed logfold changes of at least two at 1 dpi and/or 3 dpi (Fig. 3), indicating successful infection of most sampled leaflets. Not all of these changes were significant though in the corresponding pair wise comparisons of the sample groups. This can be explained by the highly variable tag counts in the individual samples (Table S2) and the asynchronous infection kinetics as shown by PCA.

DeepSAGE analysis unraveled some interesting aspects of general leaf transcriptome architecture. Two thirds (68%) of the unitags were expressed at low levels (<10 counts/million) in leaf tissue, of which one third (36%) did not match to any known potato transcript. This shows that, despite the extensive EST (expressed sequence tag) data available (DFCI Potato Gene Index), knowledge about the transcriptome composition is still incomplete, particularly in a non-model organism such as *Solanum tuberosum*. The unknown tags and their expression patterns provide a first entry point into the discovery of novel potato defense genes. The potato genome sequence [47] will provide a platform for identification of the corresponding genes. On the other hand, only 3% of the unitags showed an average expression above 100 counts/million but accounted for 32% of the total transcriptome. Of the highly frequent transcripts only 14% were unknown. The most frequent tag (StET008016, average 27184 counts/million) matched to unigene TC208859, annotated as cell wall protein. The expression of this gene accounted for 4% of all transcripts in the leaf tissues. According to the uniprot database not very much is known about the function of the encoded protein. BLASTp and motif pattern searches revealed that it belongs to the superfamily of glycine rich proteins. The potential functions of glycine rich proteins range from callose deposition and cell development to defense and stress responses and chaperone activities [48]. Not surprisingly, other highly frequent tags corresponded to genes functional in photosynthesis and primary metabolism, for example ribulose bisphosphate carboxylase, chlorophyll a–b binding proteins and other components of photosystem I and II.

Transcriptional changes during infection were much more pronounced in compatible than in incompatible interactions. The number of target sequences was however similar, 240 and 220 for incompatible and compatible interactions, respectively. The higher tag numbers in compatible interactions resulted mainly from multiple tags matching the same target sequence. Overall, the transcriptomes of incompatible and compatible interactions showed more differences than commonalities. The incompatible interaction results in programmed cell death of a small number of cells at the spot of primary contact with the pathogen. The transcriptome of these cells, which were not included in the DeepSAGE samples, might undergo dramatic changes, whereas the sampled neighboring tissue remains relatively undisturbed. The changes observed here might be causes or consequences of systemic acquired resistance (SAR) signaling [49] or of induced resistance to other types of biotic and abiotic stress called priming, which is triggered by necrotizing pathogens [50]. Three quarters of the tags differentially expressed during the incompatible interaction were not significantly up or down regulated during the compatible interaction, indicating considerable differences between the corresponding transcriptomes. Only five tags (1.3%) had an average expression above 100 counts/million in the incompatible interaction, compared with 94 (11.8%) tags in this category in the compatible interaction (Tables S3 and S4). The GO-analysis gave no hint, which cellular and metabolic processes might specifically be affected in the incompatible interaction, probably due to lack of knowledge of *Solanum tuberosum* genes functional in this context. The GO-term ‘defense response, incompatible interaction’ (GO:0009814) contained only 64 genes, while the next higher order term (GO:0006952) ‘defense response’ contained 506 genes. At present, the resolution of GO-analysis is not sufficient to identify the processes initiated or interrupted during the incompatible interaction. Consistent with this is the fact that a meaningful annotation was assigned to only one quarter of the tags showing differential expression in the incompatible interaction. Of those we discuss below two interesting cases with highly significant differential expression.

One day post inoculation, tag StET009643 matching the transaldolase gene *ToTAL2* (TC196885) was specifically and transiently up regulated during incompatible interactions (Figure 6B). Transaldolases (EC 2.2.1.2) catalyze the conversion of sedoheptulose 7-phosphate and D-glyceraldehyd 3-phosphate into D-erythrose 4-phosphate and D-fructose 6-phosphate, as part of the oxidative pentose phosphate pathway. Erythrose 4-phosphate is one precursor of the shikimic acid pathway, which gives rise to phenylpropanoids, alkaloids and the plant hormones auxin and salicylic acid (http://www.brenda-enzymes.info/php/result_flat.php4?ecno=2.2.1.2) [51]. These molecules are all associated with plant defense. Salicylic acid is an important component of SAR, in which auxins may also have a role [49]. Only two tags in the whole data set were annotated as transaldolase, one (StET009643) corresponding to tomato *ToTAL2* (AY007225) [52] and the other (StET010753) to potato *PotTAL1* (U95923) [53] and tomato *ToTAL1* (AF184164) [52]. The latter was up regulated at 3 dpi in both R1 and r1 plants but not in ORF45 plants (Figure 6A), whereby only the up regulation in r1 plants reached the significance threshold (Table S1). Up regulation of transaldolase 1 upon fungal infection has been also observed in cucumber and wheat [52], suggesting a more general role of this isoform in defense, whereas transaldolase 2 might have a more specific function in the incompatible interaction.

Three days after infection, tag StET010841 matching a *Fibrillin 8* (*FIB8*) gene (TC207935) was strongly and specifically down regulated (Table S3). Interestingly, tag StET01040, which differed by only one nucleotide, was not differentially regulated (Table S1). This tag might represent an allele or a paralogous *FIB* gene. Plant fibrillins are structural, lipid associated proteins located in thylacoid membranes, which seem to play a role in biotic and abiotic stress responses, growth and development and in hormone signaling [54]. Down regulation of Arabidopsis *FIB*'s by RNA interference resulted in reduced growth and increased symptoms of photooxidative stress when plants were exposed to a combination of high light and cold temperature. The stress symptoms could be cured by treatment with methyl-jasmonate, suggesting that FIB transcript levels condition jasmonate (JA) production, possibly by lowering the level of oleic acid-containing triacyl glycerol, a substrate for JA biosynthesis [55]. In the context of host-pathogen interactions, jasmonates are involved in SAR [49]. The specific down regulation of a potato *FIB* gene may cause decreasing JA levels around 3 dpi during the incompatible interaction. Consistent with this is the observation that down regulation of JA biosynthesis in the same *R1* transgenic Désirée lines as used here for DeepSAGE analysis did not compromise the hypersensitive response [56].

Transcriptional changes during compatible interactions are of particular interest for quantitative resistance to late blight, which is considered as natural variation of the kinetics and size of a compatible interaction. In the compatible interaction, a general reprogramming of the host tissue seems to occur, as indicated by the large number of transcriptional changes and overrepresentation of general GO terms mainly in the compatible interaction. Most tags with meaningful annotation matched gene families known to be up or down regulated during host pathogen interactions. The up regulation of pathogenesis related genes and down regulation of genes functional in photosynthesis and CO_2_ fixation during the compatible interaction is consistent with previous findings [32]. Down regulation of carbonic anhydrase, suggested to increase susceptibility to *P. infestans* [32] was represented by several tags matching the unigenes TC209461, TC218724 or TC221870 annotated as carbonic anhydrase (Table S1). Systematic comparisons with microarray experiments [31], [32], [33] are difficult at present due to differences of genetic material used, experimental set ups and the lack of common sequence identifiers. Besides the well known transcripts, DeepSAGE analysis provided also first information on novel transcripts with potential function in compatible interactions, such as down regulated TC210616, described as ‘putative 16 kD membranes protein’, up regulated TC208974, encoding an unknown protein, or the down regulated tag StET002587 without any matching sequence.

DeepSAGE allows a better dissection of the expression of multigene families. This is discussed using protease inhibitors as an example. Protease inhibitors are up regulated in response to wounding and herbivore attack. The wound response is mediated by jasmonate signaling [57]. In both compatible and incompatible interactions with *P. infestans* however, most tags matching protease inhibitors were down regulated (Table S6). This is consistent with the coordinated down regulation of Kunitz-type and Pin2 inhibitor families upon infection with *P. infestans* of two compatible cultivars, which was observed using qRT-PCR, which discriminated between inhibitor families but not between individual family members [58]. This indicates that the interaction with *P. infestans* is the contrary of a wound response, at least during the initial biotrophic phase, which might involve a decrease in jasmonate levels (see above). Some inhibitor tags however, matching certain metallocarboxypeptidase, Pin2 and ethylene-responsive protease inhibitors, were up regulated, preferentially in the compatible interaction. This suggests functional differentiation between members of some inhibitor gene families. When comparing multiple tags matching the same inhibitor sequence, large differences in average tag frequencies were evident. Multiple tags with very low frequency may be artefacts resulting from library construction and sequencing. Some may result however from variable expression of individual members of the gene family and/or allelic variants, which became visible for the first time in the DeepSAGE data.

The DeepSAGE experiment allowed to assess the effect on the leaf transcriptome of the presence of the transgenes *R1* and *ORF45* in cv Désirée background. Seven tags were expressed at higher or lower level at 0 dpi in the R1 plants as compared with the r1 and ORF45 plants (Table S7). These seven tags might be the consequence of the presence or absence of the *R1* transgene in the genome of cv Désirée, or they might have a role in the very early onset of incompatible versus compatible interactions (the 0 dpi samples were collected immediately after inoculation, see Materials and Methods). The only tag with meaningful annotation matched *GRAS9* (TC210616), a member of GRAS transcription factor family. This transcript was undetectable in the *R1* transgenic plants at 0 dpi, whereas it was detected in moderate levels in r1 and ORF45 plants. One and three days after infection, the transcript was present at low level in all plants. GRAS transcription factors are known to be involved in developmental processes, but may also have a role in host pathogen interactions. GRAS transcripts accumulated in tomato during incompatible interactions and silencing a member of this family (SIGRAS6) impaired tomato resistance to bacterial speck disease [59]. Although speculative at this point, our findings point to a possible connection between the presence of NBS-LRR type resistance genes such as *R1* and repression of GRAS transcriptional regulators.

The ORF45 transgenic line was included in the DeepSAGE analysis with the aim to capture differential transcripts caused by the presence of the *r1.1* transgene, which is physically closely linked and highly similar to the *R1* resistance gene [60] but does not confer resistance to *P. infestans* [6]. Transcripts specifically up or down regulated in ORF45 plants might hint at a possible function of the *r1.1* gene that is independent from pathogen resistance. We could not obtain evidence however, that the ORF45 transgenic line contains a full length copy of the *r1.1* transgene. Differences observed between the leaf transcriptomes of ORF45 and wild type plants can therefore result from unspecific effects of transgene copy number and position, and/or from specific effects of functional sequences present in the transgenic DNA insertion(s) in ORF45 plants. A surprisingly large number of tags were present in higher frequency in ORF45 plants at 0 dpi when compared with R1 and r1 plants. Part of the reason for this might be that in case of ORF45 only three instead of six samples were available for comparisons, which reduced the reliability of the comparison. An unknown proportion of significant tags likely resulted from random biological variation of transcript levels. On the other hand, some transcriptional differences might be the consequence of the transgenic DNA insertion(s) in ORF45 plants. Interestingly, many tags overrepresented in ORF45 plants at 0 dpi were matching genes for photosynthesis, CO_2_ fixation and respiration. Phenotypically, there was however no visible difference between non infected ORF45, R1 and r1 plants.

### Conclusion

Using DeepSAGE technology for the generation of transcriptome data and subsequent computational data analysis delivered a suite of novel candidate genes that might play a functional role in compatible and incompatible host pathogen interactions in general and interactions of potato with *Phytophthora infestans* in particular. Further research is required to elucidate their putative role in more detail. The tag matching unigenes and their annotation provide the entry points for structural and functional exploration of these genes, for example by gene silencing or association studies. For the ‘unknowns’, the potato genome sequence [47] provides a promising new tool for identifying the corresponding genes. The set of data described in this paper can be used as an experimental basis for the development and application of novel bioinformatics tools, which might lead to further insights in the biological process of host pathogen interactions in plants.

## Materials and Methods

### Plant material

For comparative transcriptome analysis of the compatible versus the incompatible interaction with *P. infestans* we used the cultivar Désirée and four transgenic Désirée lines. The independent transgenic lines 10-2/4 and 10-23/2 both carried the *R1* resistance gene and showed the hypersensitive response upon infection with P. *infestans* races having the *Avr1* gene (incompatible interaction) [6]. Progeny tests of both lines had shown that *R1* was inherited as a single locus [61]. Untransformed Désirée (WT) and a transgenic line (LV41) transformed with the empty vector pCLD04541 [62] used for complementation analysis of *R1* [6] were used for the compatible interaction. The fourth transgenic line (ORF45) has been transformed with a 10 kbp genomic fragment containing the *r1.1* gene [6], which corresponds to ORF45 in [60]. Agrobacterium mediated transformation was performed as described [6]. Line P4H5K3S2 shows a compatible interaction with *P. infestans* races having the *Avr1* gene. The presence of the left border, but not the right border of the 10 kbp insertion could be demonstrated in line P4H5K3S2 by PCR using primers that flank the vector-insertion junction (not shown). Primers designed for specific detection of ORF45 failed to discriminate between the transgene and endogenous members of the *R1* gene family present in cv Désirée. Copy number and integrity of the *r1.1* transgene in line P4H5K3S2 are therefore unknown. Transgenic and wild type Désirée plants were regenerated from in vitro shoot cultures and grown under long day conditions (16 h light, 80 mmol photons m^−2^ s^−1^, and 8 h darkness) in a climate chamber (York International, Mannheim, Germany) at 21°C.

### *P. infestans* cultivation and inoculum preparation

*Phytophthora infestans* strain R208m^2^ race 4 (*Avr1*) [63] was propagated either sterile at 18°C on rye agar plates [64] containing small leaves from susceptible in vitro grown potato or non-sterile in a Petri dish on detached leaflets from 6–10 week old susceptible plants. Long term cultures were maintained at 12°C. The race composition was controlled by a detached leaflet test [65] using the race 1–11 differential set of potato cultivars from the Scottish Crop Research Institute (Invergowrie, UK). For inoculum preparation, sporangia were collected in deionized water from 6–11 days old infected leaves. Sporangia concentration was quantified in a Neubauer chamber and adjusted to the required concentration. Sporulation was induced by incubating the sporangia suspension at 4–8°C for 3–6 h. Successful sporulation was controlled optically using a Stereomicroscope. Detached leaflets were inoculated with 20 µl sporangia suspension (30000–60000 sporangia/ml). Infection symptoms were observed between the fifth and the 7th day post inoculation and confirmed by the presence of mycelia.

### Infection experiments

Plants were grown for 6–8 weeks under standard conditions. At least one week before infection, the plants were transferred to a growth chamber (Vötsch Industrietechnik, Balingen, Germany) with lower temperature (16 h light at 18°C, 8 h dark at 14°C). Four leaves of each plant were used for infection starting at the third leaf from the top. Each terminal leaflet was infected with four 12.5 µl droplets of inoculum (30 000 sporangia/ml). The droplets were placed in the four corners of the leaflet. Tissue samples were collected by punching out a leaf disc of 2 cm in diameter from the center of the leaflet, excluding the inoculation spots. The discs of three leaflets derived from one plant were pooled, immediately frozen in liquid nitrogen and stored at −80°C. The fourth inoculated leaflet was harvested eight days post inoculation to verify a successful infection by semi-quantitative amplification of genomic *P. infestans* DNA using the primers 0–83 and 0–84 [66] and as template genomic DNA extracted from the whole leaflet. Inoculation and harvest of leaf discs were done in the late afternoon (circa 16:30 to 20:00 o'clock) at zero (0 dpi), one (1 dpi) and three (3 dpi) days post inoculation. The 0 dpi samples were collected immediately after inoculation with a contact time of less than three minutes. The plants were then covered with clear plastic bags to insure high humidity. For each time point material was harvested from a different plant to avoid wounding and priming effects. The infection experiment was repeated three times with new batches of plants and inoculum (Rep1, Rep2 and Rep3).

### 3′-tag library production and Deep sequencing

Total RNA was extracted from infected leaf tissue using the ToTALLY RNA Kit (Applied Biosystems, Carlsbad, USA) following the suppliers instructions. RNA was quantified using the Nanodrop system of Thermo Scientific (Waltham, USA). Prior to the production of 3′-tag libraries the RNA was assessed for integrity on 1% agarose gel by the presence of intact ribosomal RNA bands. Two µg of total RNA per sample was used to construct DeepSAGE tag libraries [36] using a modification to facilitate direct sequencing of the amplicons by Solexa/Illumina sequencing [67]. Samples were diluted to a final concentration of 10 nM and were pooled into four pools each containing 10 samples with different identification key. Final pool concentrations were estimated using Quant-iT-PicoGreen prior to template DNA hybridization and sequencing on an Illumina Genome Analyzer II (Illumina, San Diego, USA) according to the manufacturer's instructions. Due to the restriction enzyme *Nla*III used for library construction, each tag carries the nucleotides CATG at the 5′ end, which were not included in the tag sequences shown in the supplementary tables.

### Data analysis

The tag count data were transformed into a data matrix using a series of Perl-scripts available for download on the PoMaMo database [68] (http://www.gabipd.org/projects/Pomamo/). Sequence tags detected less than twice (singletons) were removed from the data using the script “CutoffLibsV3.pl”. Then the data of all libraries were combined in one table using “CompareSage.pl”. The tag count data was transformed into the relative unit counts/million by implementing “NormaliseTagTable.pl”. To this data matrix a second cutoff was applied using “CutoffCombinedLibsTable.pl”. This cutoff was defined as a minimum of 110 tag counts/million summed up over all libraries and a minimum detection in three libraries. As last step the tag annotation was performed by the implementing “GlobalSageMap-V23.pl”. The source for the tag annotation was a combined reference sequence set consisting of the currently available Potato Gene Index “Potato 13.0” (http://compbio.dfci.harvard.edu/tgi/plant.html from the Dana Faber Cancer Institute) and the genomic sequence of *Phytophthora infestans* T30-4 (http://www.broadinstitute.org/annotation/genome/phytophthora_infestans/MultiHome.html at the Broad Institute) [37]. Taking into account the high sequence diversity and SNP (single nucleotide polymorphism) density within *Solanum tuberosum* [69] one nucleotide mismatch was allowed for a successful tag annotation.

Logfoldchange (logarithm 2 of the fold-change of a tag count between two samples), p and FDR values were calculated using the software R v.2.11.1 in combination with the extension package edgeR 1.6.5 [46]. For the calculation of p-values the tag wise dispersion was estimated using a grid length of 1000. P values were corrected for multiple testing with the method after Benjamini & Hochberg [70]. Comparisons were made between grouped samples (groups R1, r1 and ORF45, Table 1) at a given time point or between a single group at different time points.

Principal component analysis was performed using the princomp command included in R v.2.11.1. and the normalized expression data matrix.

Hierarchical cluster analysis was performed using the online services CARMAweb v.1.5 and GenesisWeb v.1.1 (https://carmaweb.genome.tugraz.at/carma/ and https://carmaweb.genome.tugraz.at/genesis/index2.html) using average linkage clustering and Spearman rank correlation.

GO-Term analysis was performed using tags which showed a significant expression change (FDR≤0.1) at three days post inoculation compared to 0 days post inoculation from the same group. The software Cytoscape v.2.6.3 in combination with the extension Bingo v.2.3 [71] was used for the analysis. The mapping source was the currently available information from the Dana Faber Cancer Institute for *Solanum tuberosum* (http://compbio.dfci.harvard.edu/cgi-bin/tgi/gimain.pl?gudb=potato), which was adapted to the needs of the software (the modified version will be available at the PoMaMo database). Fishers Exact test was used in combination with the multiple testing correction according to [70]. Significance of overrepresentation (FDR≤0.05) was calculated related to the whole annotation information. FDR≤0.05 was accepted as significant. The network diagram of overrepresented GO-terms was created using AMIGO v.1.7 (available under http://amigo.geneontology.org/cgi-bin/amigo/go.cgi) [72].

## Supporting Information

Table S1 **Tag identification, sequence, annotation and normalized tag counts (Columns X to BO) of 30.859 unitags; log-fold change values (logFC) and false discovery rates (FDR) of the nine pair wise comparisons (columns E to V) between sample groups (** **Fig. 4** **).** FDR values<0.05 are highlighted white.
(XLSX)

Table S2 **Tag identification, sequence, annotation and normalized tag counts (Columns X to BO) of 146 ‘defense response’ (GO:0006952) unitags; log-fold change values (logFC) and false discovery rates (FDR) of the nine pair wise comparisons (columns E to V) between sample groups (** **Fig. 4** **).** FDR values<0.05 are highlighted white.
(XLSX)

Table S3 **Tag identification, sequence, annotation and normalized tag counts (Columns X to BO) of 390 unitags differentially regulated (FDR<0.05) only in incompatible interactions; log-fold change values (logFC) and false discovery rates (FDR) of the nine pair wise comparisons (columns E to V) between sample groups (** **Fig. 4** **).** FDR values<0.05 are highlighted white.
(XLSX)

Table S4 **Tag identification, sequence, annotation and normalized tag counts (Columns X to BO) of 796 unitags differentially regulated (FDR<0.05) only in compatible interactions; log-fold change values (logFC) and false discovery rates (FDR) of the nine pair wise comparisons (columns E to V) between sample groups (** **Fig. 4** **).** FDR values<0.05 are highlighted white.
(XLSX)

Table S5 **Tag identification, sequence, annotation and normalized tag counts (Columns X to BO) of 28 unitags differentially regulated (FDR<0.05) in both compatible and incompatible interactions; log-fold change values (logFC) and false discovery rates (FDR) of the nine pair wise comparisons (columns E to V) between sample groups (** **Fig. 4** **).** FDR values<0.05 are highlighted white.
(XLSX)

Table S6 **Tag identification, sequence, annotation and normalized tag counts (Columns X to BO) of 150 unitags annotated as protease inhibitors; log-fold change values (logFC) and false discovery rates (FDR) of the nine pair wise comparisons (columns E to V) between sample groups (** **Fig. 4** **).** FDR values<0.05 are highlighted white.
(XLSX)

Table S7 **Tag identification, sequence, annotation and normalized tag counts (Columns X to BO) of 7 unitags differentially regulated (FDR<0.05) in incompatible versus compatible interactions at 0 dpi; log-fold change values (logFC) and false discovery rates (FDR) of the nine pair wise comparisons (columns E to V) between sample groups (** **Fig. 4** **).** FDR values<0.05 are highlighted white.
(XLSX)

Table S8 **Tag identification, sequence, annotation and normalized tag counts (Columns X to BO) of 1471 unitags differentially regulated (FDR<0.05) only in compatible ORF45 interactions; log-fold change values (logFC) and false discovery rates (FDR) of the nine pair wise comparisons (columns E to V) between sample groups (** **Fig. 4** **).** FDR values<0.05 are highlighted white.
(XLSX)

Table S9 **Tag identification, sequence, annotation and normalized tag counts (Columns X to BO) of 199 unitags differentially regulated (FDR<0.05) in ORF45 versus R1 and r1 at 0 dpi; log-fold change values (logFC) and false discovery rates (FDR) of the nine pair wise comparisons (columns E to V) between sample groups (** **Fig. 4** **).** FDR values<0.05 are highlighted white.
(XLSX)
